# Research on complex air leakage method to prevent coal spontaneous combustion in longwall goaf

**DOI:** 10.1371/journal.pone.0213101

**Published:** 2019-03-01

**Authors:** Kun Wang, Haibo Tang, Fengqi Wang, Yong Miao, Dapeng Liu

**Affiliations:** 1 College of Mining Engineering, Taiyuan University of Technology, Taiyuan, Shanxi, China; 2 Sitai Coal Mine, Datong Coal Mine Group, Datong, Shanxi, China; 3 Chengde Petroleum College, Chengde, Hebei, China; Central South University, CHINA

## Abstract

Spontaneous combustion of coal is one of the major hazards threatening production safety during longwall mining. Mining-induced voids, which provide passages for air leakage, are the key factor triggering spontaneous combustion of coal in longwall goafs. In this study, a comprehensive method, which combined pressure balance, grouting injection, and filling fissures, was proposed to prevent spontaneous combustion of coal in longwall goafs with complex air leakage. Field engineering practice was carried out in Sitai Coal Mine in China. The results demonstrated that with the application of the proposed method, in the working face, the concentration of CO was decreased from 31ppm to 0 and the air leakage quantity was decreased from 261 to below 80 m^3^min^-1^. The gas samples analysis from the gob areas also indicated that concentrations of O_2_ and CO were successively decreased, indicating that the risk of spontaneous combustion of coal in goafs was eliminated. The above mentioned analysis indicates that, the method proposed in this study is useful and efficient. Successful application of this technology could provide reference for the treatment of other coal mines.

## Introduction

China is the largest coal-producing and consuming country in the world, and 90% of coal comes from the longwall mining faces [1, 2]. During longwall mining, the underground coal fires are a major hazard threatening production safety [3–5]. Majority of underground coal fires are caused by spontaneous combustion of coal, and the longwall gob area is one of the main places that is prone to spontaneous coal combustion [6]. Coal fires occurring in goafs not only produce large amounts of toxic and harmful gases but also cause production interruption on the working face [7]. Therefore, the research on spontaneous combustion in longwall goafs has always been an important global issue [8].

One of the main reasons for the spontaneous combustion of coal in longwall gob areas is mining-induced air leakage passages [9]. During coal mining, the overlying strata will cave, fracture and subside with the expansion of the gob area [10], while the ground surface may collapse and crack [11]. The fracture and movement of strata produce many voids, including pores among the rubble, fractures in the strata, and fissures in the ground, which can act as air leakage passages that allow air penetration [12–13]. Furthermore, the oxygen present in the atmosphere and underground ventilation can enter the gob areas through these voids and interact with the residual coal in the goaf. Oxidation of coal liberates heat, which on accumulation, leads to the increase in temperature [14–15]. When the temperature reaches the ignition point, the coal starts to burn, and this may cause the spontaneous combustion as time passes [16–17]. These voids that serve as air leakage passages in the mining-disturbed strata, constitute the key factors that affect the generation of spontaneous combustion in longwall gob areas [18].

Extensive research efforts have been devoted to the prevention of spontaneous combustion of coal in longwall goafs. Zhou et al. applied the three-phase foam, which was composed of mud, nitrogen and water, to confront an extraordinarily severe coal mine fire [19]. G.J. Colaizzi developed a technology through cellular (foam containing) grout to mitigate coal fires in the gob area from the perspectives of prevention, control and extinguishment [20]. Liang and Wang proposed three numerical models for the solution of fluid dynamics and heat transfer issue in both longwall face and goaf, to evaluate the risk of the coal mine goaf self-heating hazard [21]. Lu et al. analyzed the oxygen concentration distribution with Fluent, successfully applying the foam slurry technology to prevent and control spontaneous combustion of coal [22]. Brune and Saki discovered that, for progressively sealed gobs, a targeted injection of nitrogen from the headgate and tailgate would create a dynamic seal of nitrogen to effectively prevent the gob ignitions [23]. In contrast, studies on the prevention of spontaneous combustion of coal in longwall gob area with complex air leakage passages have rarely been reported. Under the complex air leakage conditions, the air leakage passages are unpredictable, and the air leakage sources are complex. Moreover, a higher number of mining-induced air leakage passages appears when longwall face advances forward [24]. Current methods, such as nitrogen injection and grouting, cannot effectively control the air leakage passages or prevent the gas exchange among the gob areas and the working face caused by the complex air leakage. Even if the goaf fire is extinguished, with the emergence of new leakage passages, a spontaneous combustion might also occur again.

In this study, the field analysis and investigation of the mining-induced air leakage passages were described in detail. Furthermore, the prevention method on of spontaneous combustion of coal in longwall gob areas caused by these air leakage passages was proposed. Field engineering practice was also carried out in Sitai Coal Mine in China.

## Description of the coal mine study

**Fig 1:**
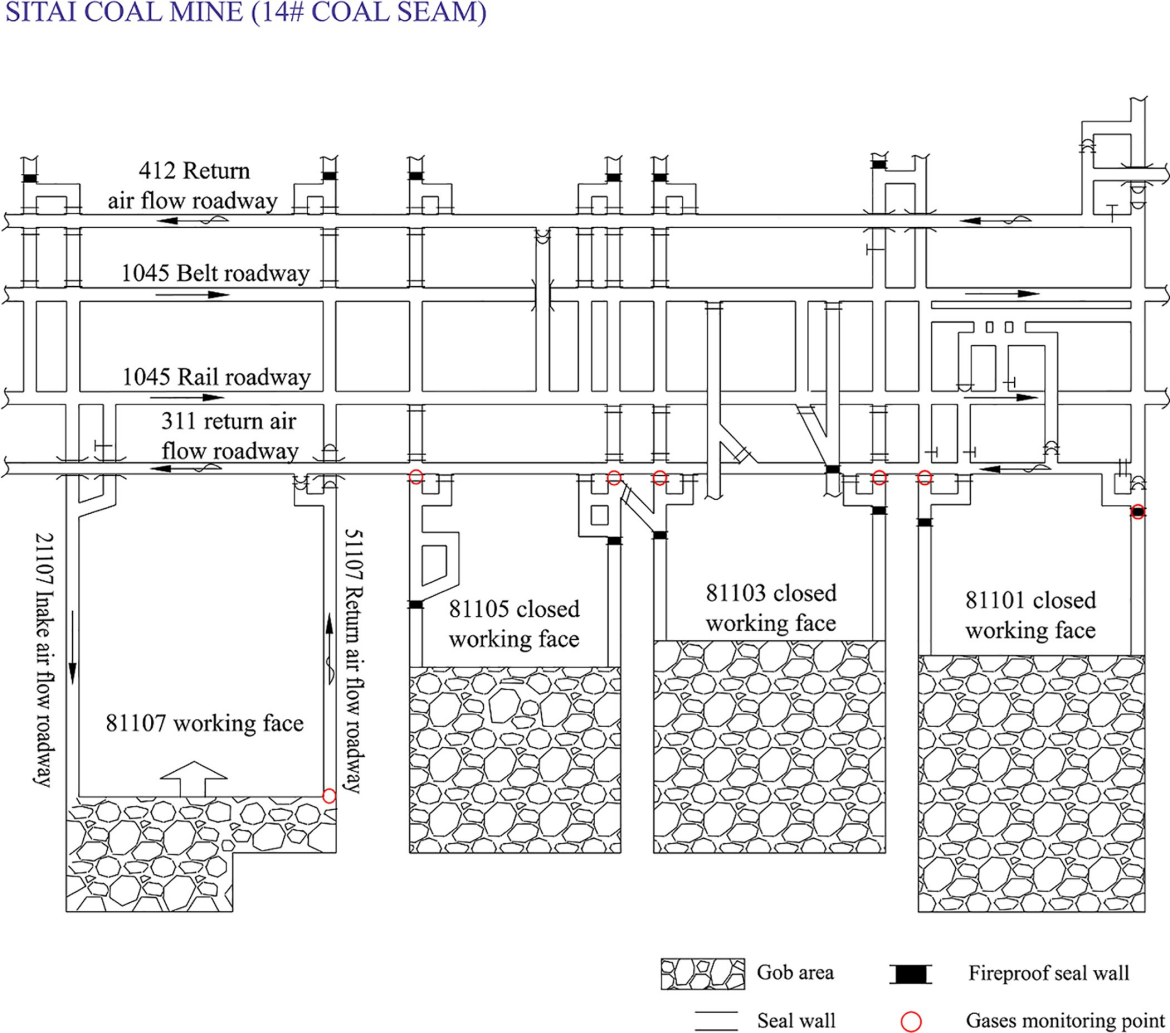
Description of 311 mining area in Sitai Coal Mine.

Sitai Coal Mine is located at the western Datong Basin of Shanxi province in China. It covers an area of 65.45 km^2^. The coal production is 4.2Mt/a. The mine belongs to high gassy coal mine and the negative pressure ventilation has was adopted. The main minable coal seam was the 14# coal seam. The 12# coal seam is the overlying seam and the distance between them is only 8.3–15.7m, thus the gobs of two coal seams interconnect with each other. Large amounts of harmful gases were gathered in 12# coal seam gob areas, which brought danger of spontaneous combustion of coal to 14# coal seam. In order to prevent fresh air from entering the gob areas and to detect the harmful gases, all gob areas of 14# coal seam were sealed with fire-proof closed walls, and seven gas monitoring points were set up, as presented in Fig 1.

The 81107 longwall working face in 14# coal seam, which belongs to 311 mining area, is currently mined, with an average operating depth of 293 m. Mining leads to the destruction of the surrounding rock and coal seam. A large number of complex and unpredictable voids passages emerged in the broken rock around the 81107 working face and its surrounding gob areas. These voids can provide the passages for gas exchange among the gob areas and the working face. Noxious gases, such as CO and CH_4_, could flow into the working face by passing through these passages under the action of negative pressure ventilation. Moreover, fresh air from working face would enter the gob areas, resulting in the hidden danger of increase of spontaneous combustion of coal. Table 1 summarizes at the upper corner of 81107 working face, the concentrations of CO and CH_4_ increased, whereas the O_2_ content decreased as the mining progressed. On the third day of mining, the concentration of CO reached 31ppm, exceeding the alarming concentration (24ppm). Moreover, the air quantity difference between the intake air and the return air of the working face increased from 63 to 261 m^3^min^-1^, which indicated that the quantity of air entering the gob areas also increased. The air quantity monitors were placed in the intake and return airflow roadways of the 81107 working face, 30 m away from the 311 return airflow roadway. These problems not only led to the temporary shutdown of the 81107 working face, but also led to the exacerbated risk of spontaneous combustion of coal.

**Table 1:** Variations of gases concentration and air leakage quantity of 81107 working face as mining progressed.

Mining date (days)	Gases concentration at upper corner			Intake air quantity (m^3^min^-1^)	Return air quantity (m^3^min^-1^)	Air quantity difference (m^3^min^-1^)
	CH_4_ (%)	CO (ppm)	O_2_ (%)			
**1**	0	0	20	923	986	63
**2**	0.23	6	19.7	845	972	127
**3**	0.71	31	18.2	730	991	261

Considering the aforementioned problems, necessary measures should be taken to solve them. Furthermore, the air leakage passages of Sitai Coal Mine were unpredictable, and the air leakage sources were complex, while the goaf area effect by air leakage was high; therefore, the commonly used methods, such as nitrogen injection and grouting, were not effective. Therefore, it was necessary to put forward a comprehensive method to control the spontaneous combustion of coal in longwall goafs with complex air leakage.

## Analysis of air leakage passages

### Air leakage passages connecting ground, 12# coal seam and 81107 working face

With the advancement of working faces, the overlying strata formed caved zone, fractured zone, and continuous zone [25–26]. Gas could flow within the caved zone and the fractured zone through vertical fractures [27]. If the coal seam was not deep enough or there were other elements capable of promoting the formation of the fractured zone, the vertical fractures would interconnect the ground surface, so that the fresh air could flow into the caved zone and the gob. According to Palchik [28], the fractured zone is divided into three parts: rock blocks, through-going vertical fractures, and horizontal fractures. The vertical fractures are interconnected by horizontal fractures, which promote the formation of air leakage passages.

**Fig 2:**
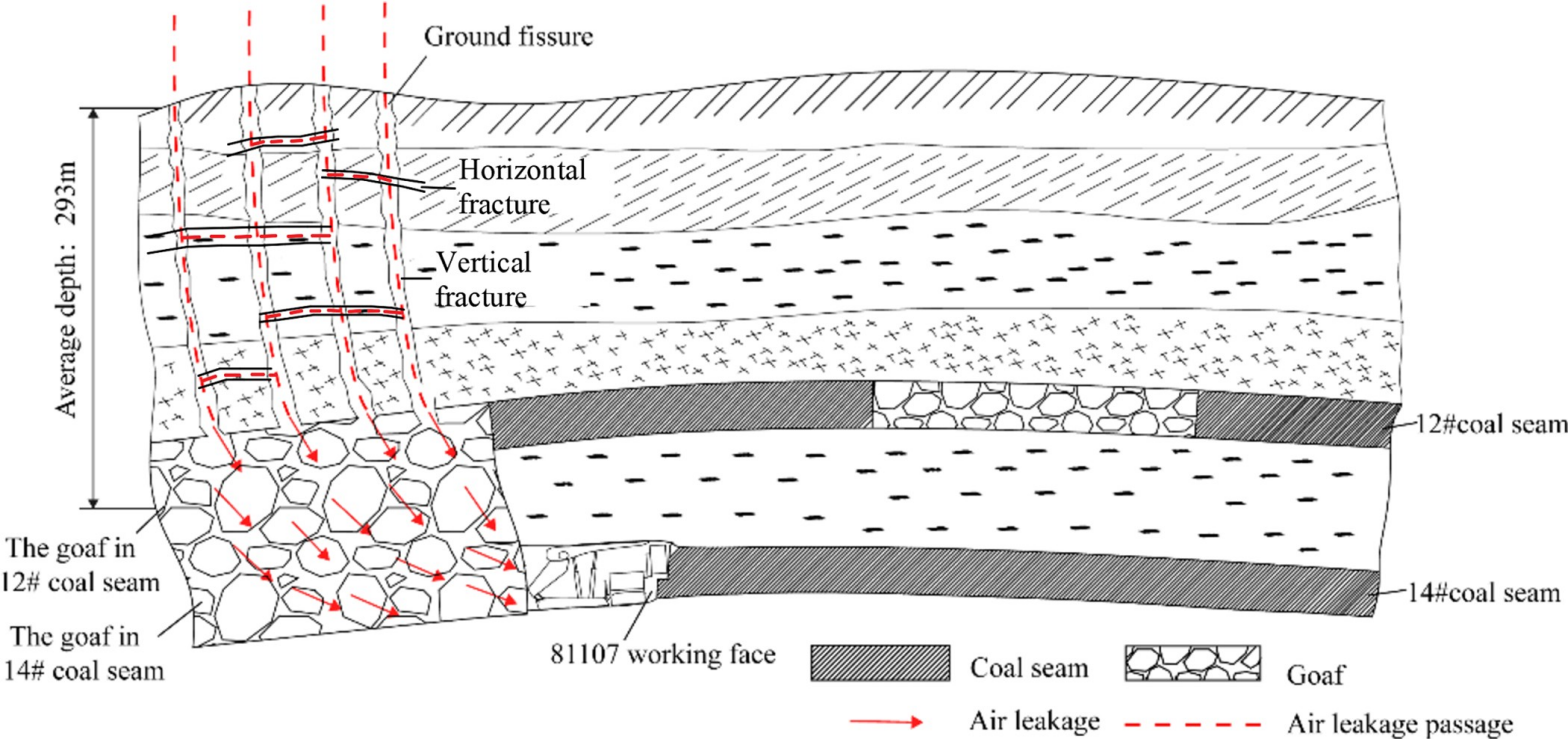
Schematic illustration of air leakage passages connecting ground, 12# coal seam and 81107 working face.

Fig 2 presents the schematic illustration of air leakage passages connected to the surface, as well as the 12# coal seam and 81107 working face. The fractured zone of 311 mining area interconnected the gob of 12# coal seam, and the vertical fractures interconnected the caved zone and the fractured zone of 12# coal seam. Furthermore, the vertical fractures of 14# coal seam were interconnected by the horizontal fractures of 12# coal seam, as presented in Fig 2. Moreover, the ground surface collapsed and cracked. In the above mentioned processes, the fracture and movement of strata produced many voids, including vertical and horizontal fractures in the strata and fissures in the ground. The mining depth was low, and the distance between the 14# and the 12# coal seams was low; therefore, most of these voids usually extended from the underground to the ground, providing the air leakage passages connecting the ground, the 12# coal seam and the 81107 working face.

**Fig 3:**
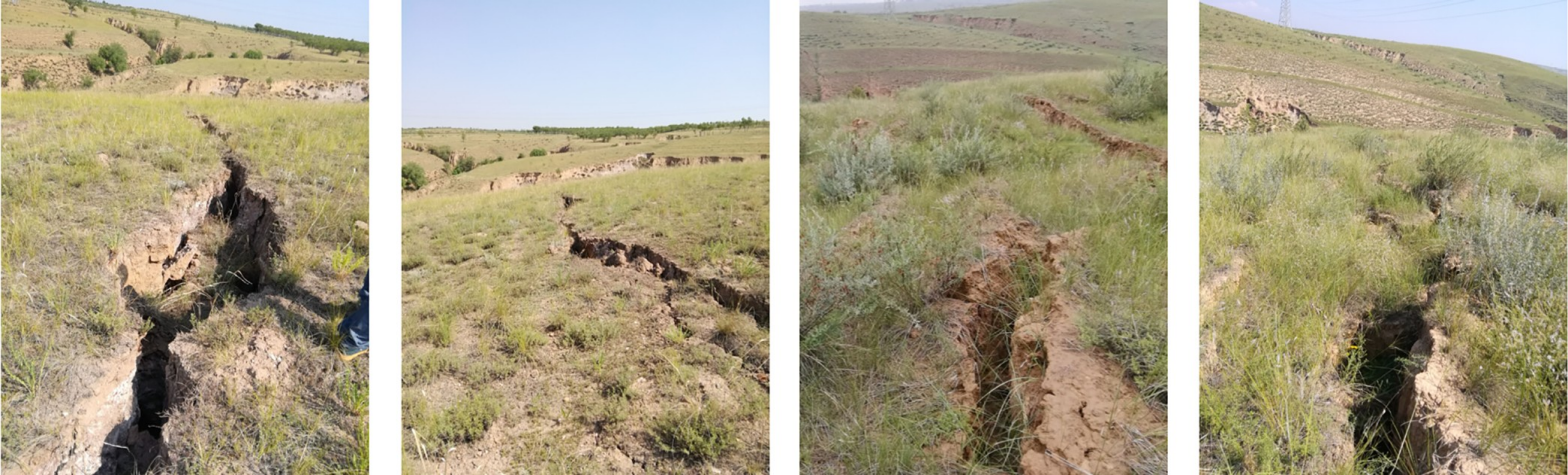
Fissures on 81107 working face ground.

Fig 3 illustrates the field investigation, exhibiting that 12 fissures were found on the 81107 working face ground and four fissures were formed subsequently to the advancement of working face. The length of these fissures was tens to hundreds of meters and the maximum width was 2m. They extended to the underground and the width gradually decreased from top to bottom with an average depth of 293m. Release of smoke on the ground proved the existence of air leakage passages from ground to 81107 working face, and there were air leakages for oxygen flow into the gob.

### Air leakage passages connecting 81107 working face and adjacent mine

**Fig 4:**
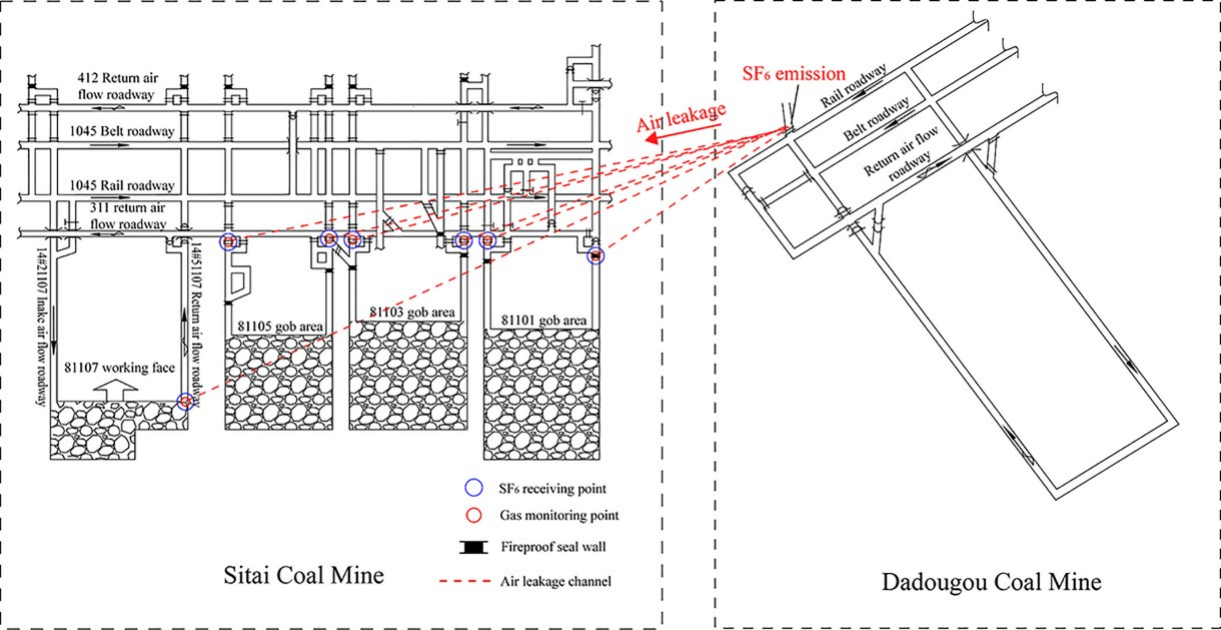
Schematic illustration of air leakage passages connecting 81107 working face and adjacent Dadougou Coal Mine.

According to Palchik [28], in the caved zone, fractures between irregular shapes of the collapsed immediate roof are interconnected. Therefore, air leakage passages are formed between adjacent coal mine gob areas. Fig 4 presents the schematic illustration of air leakage passages connecting the 81107 working face and the Dadougou Coal Mine. Dadougou Coal Mine is adjacent to Sitai Mine. It has positive pressure ventilation, while the Sitai Mine has negative pressure ventilation, which causes high air pressure difference between the two mines. With the progress of the 81107 working face, a high amount of mining-voids appears. Under the action of air pressure difference, the air leakage from the gob area of Dadougou to the 81107 working face occurs.

Fig 4 exhibits that SF_6_ as the tracer gas was released at the sealed gob area of Dadougou Coal Mine, 2500 m away from the 81107 working face. 15 minutes later, SF_6_ was received at the 81101, 81103, and 81105 gob areas and the 81107 working face. This indicated the existence of air leakage passages between the Dadougou Mine and the Sitai Mine.

Based on the latter analyses, it could be observed that two main types of air leakage passages affecting the Sitai Coal Mine are: (1) The air leakage passages connecting the surface, the 12# coal seam and the 81107 working face, and (2) The air leakage passages connecting the Sitai Mine and Dadougou Mine. These air leakage passages had their own characteristics, including complex and unpredictable sources, as well as highly affected gob areas. Furthermore, as the mining progressed and the gob areas expanded, new air leakage passages appeared.

### Method of prevention of spontaneous combustion of coal

Complex multi-source air leakage passages and gases exchanged among the gob areas and the working face under the action of ventilation, were mainly responsible for the risk of spontaneous combustion of coal in goafs and the temporary shutdown of coal production. A comprehensive method was proposed to control the spontaneous combustion in goafs and recover the coal production. Initially, the gas exchange among the gob areas and the working face was inhibited through the pressure balance method, in which, the pressure difference between the ends of air leakage passages was reduced. Slurry grouting into the sealed goafs was consequently adopted, in order to decrease the amounts of harmful gases and reduce the contact surface between coal and O_2_, thus prolonging the spontaneous combustion period of coal. Moreover, the ground fissures were filled to reduce the fresh air flow from the ground into the underground goafs.

### Pressure balance in 81107 working face

Pressure balance is a method to change the pressure distribution of the working face through the setup of pressure regulating devices, to reduce the pressure difference between the working face and the goaf, to limit the gas flow exchange between them. According to the field pressure measurement, the pressure difference between the goaf and the working face was approximately 700–750 Pa as compared to prior pressure balance. Therefore, through the consideration of site conditions of the roadway layout of the 81107 working face, the air doors and the local fans were set to perform a pressure increase of the working face by approximately 750 Pa.

**Fig 5:**
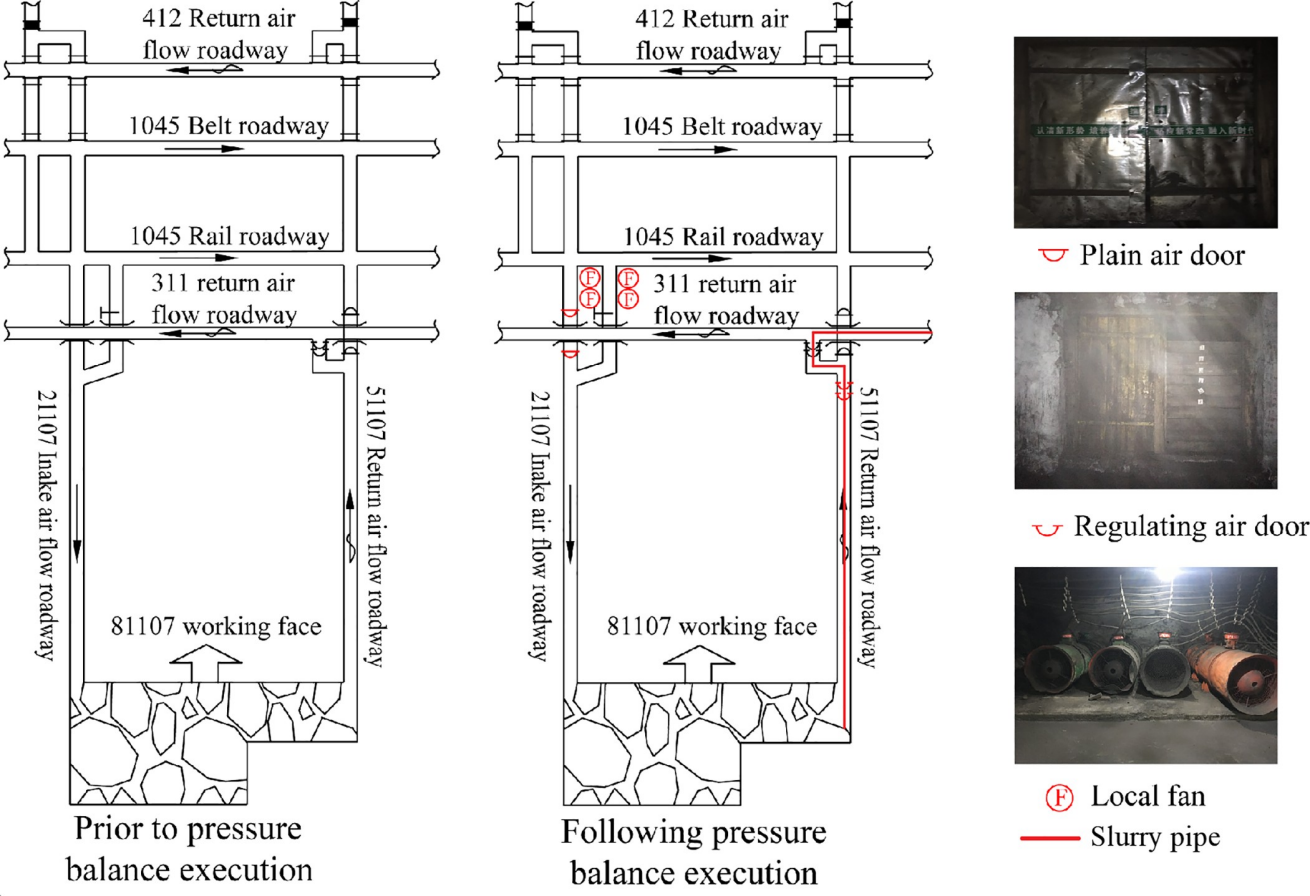
Schematic illustration of pressure balance system in 81107 working face.

Fig 5 presents a schematic configuration of the pressure balance system in the 81107 working face. The pressure balance equipment mainly consisted of four local fans, two regulating air doors and two plain air doors. First, the two air doors were set in the 21107 intake airflow roadway to block the intake air flow from the belt and the rail roadway. Subsequently, four local fans (including two standby local fans) were set in the branch of the 21107 intake airflow roadway to provide the power for the pressure increase of the working face. The pressure adjustment was achieved by the variable area change of the regulating air doors, which were installed in the 51107 return airflow roadway.

The pressure balance could effectively inhibit the air flow exchange between the goaf and the working face, as well as reduce the concentrations of harmful gases in the working face and oxygen supply for the gob areas. Adversely, because the area affected by pressure balance was much smaller than that of the entire goaf, and a high amount of accumulated harmful gases existed in the goaf; therefore, the hidden danger of spontaneous combustion in goaf was not completely eliminated. As a result, the method of goaf grouting should be further adopted, to prevent the accumulation of harmful gases in the goaf.

### Ground grouting in gob areas

Ground grouting stations, including the centralized grouting stations and two scattered grouting stations, were established at Sitai Coal Mine. The centralized grouting station was on the ground of near 81105 working face, and the boreholes of centralized grouting stations were connected to the underground slurry pipelines in 311 return airflow roadway. The slurries flowed into the each goaf from the centralized grouting stations through slurry pipes. The scattered grouting stations were on the ground of goaf area, and the boreholes were directly connected to the middle of goaf area. There were three boreholes in total. The slurries could be directly grouted into the goafs.

**Fig 6:**
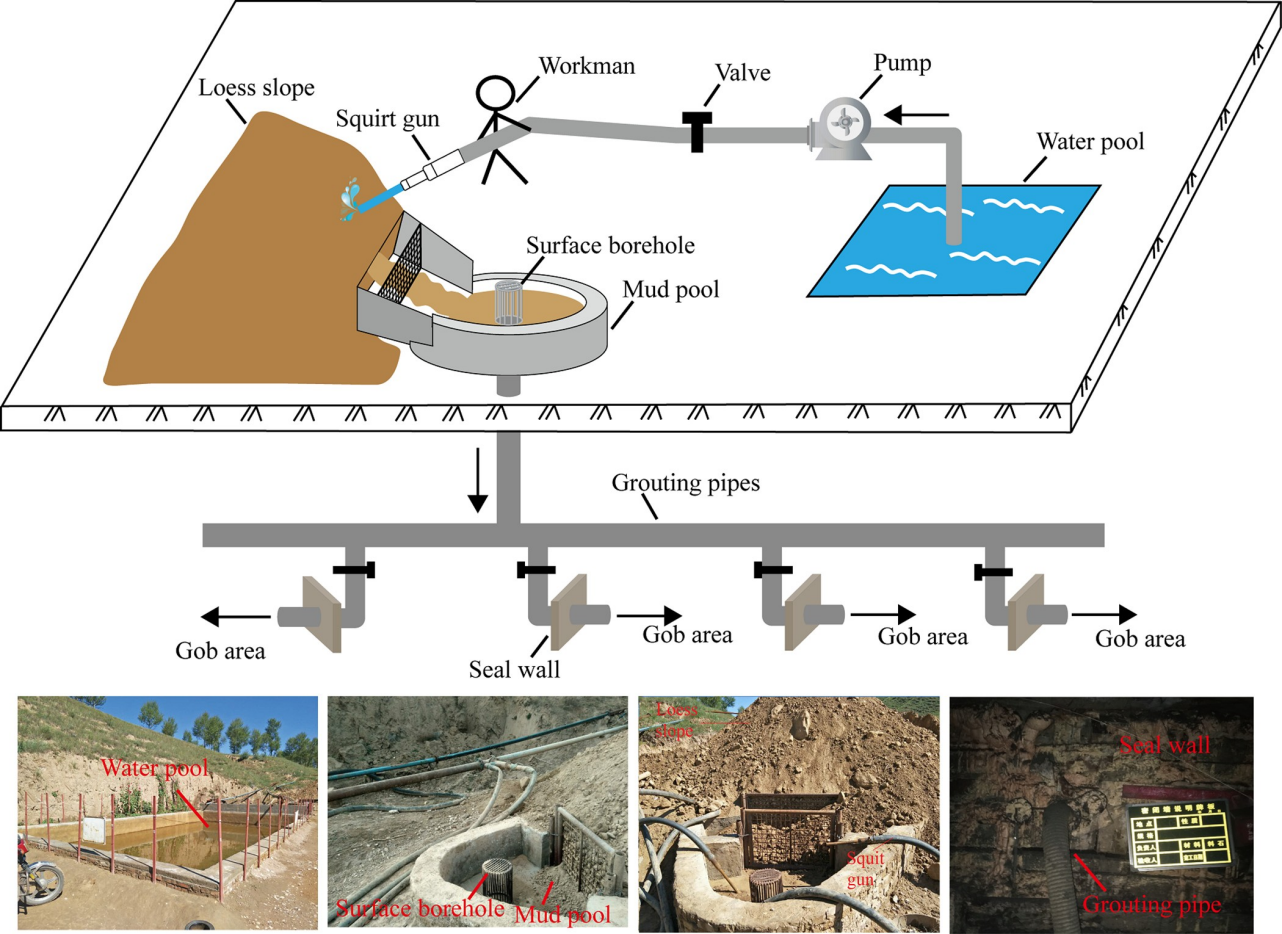
Flow diagram of slurry preparation and transportation.

The preparation and transportation processes of the slurries of the centralized grouting stations are presented in Fig 6. The specific technological procedures are as follows: the initial slurry was composed of water and loess. First, the water was pumped from the water pool into the squirt guns and sprayed to the loess slope, as well as mixed with the loess to form the slurry. Consequently, the slurry flowed into the mud pool passing by the surface borehole, which was set in the mud pool to enter the slurry pipelines of the underground mine. The slurry was subsequently delivered to the goaf along the slurry pipelines. The ends of pipelines were connected to the goaf seal walls.

**Fig 7:**
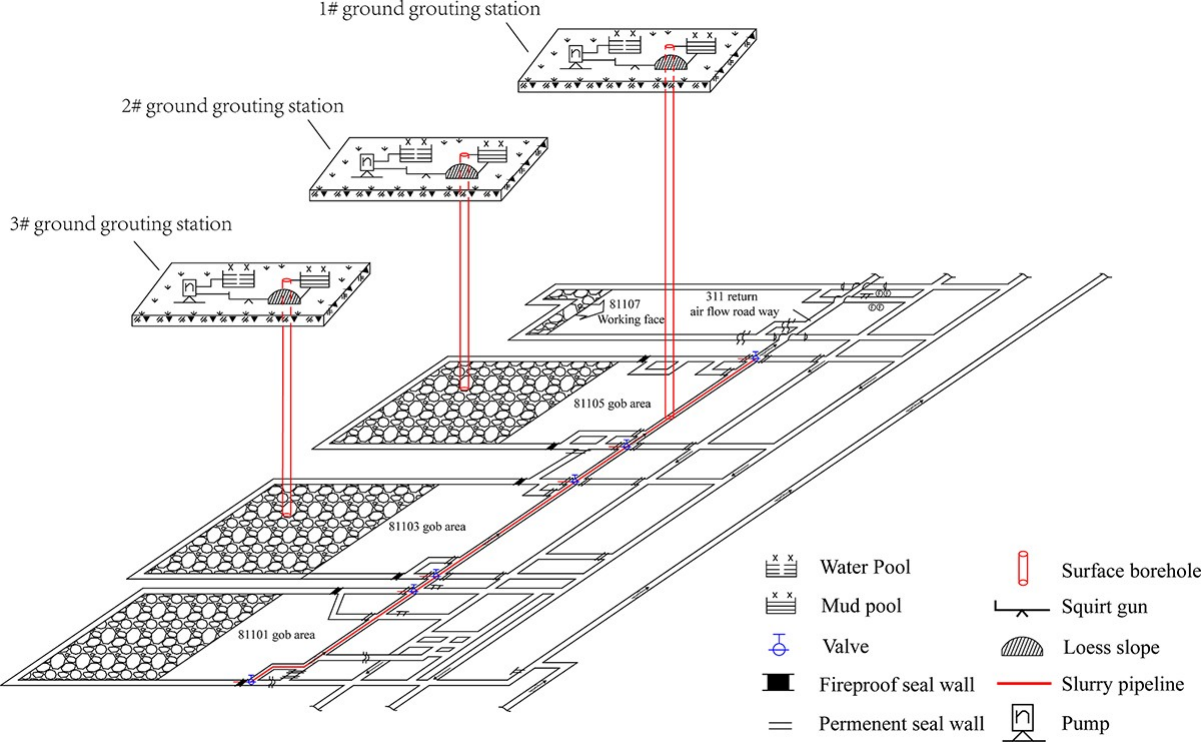
Diagram of ground grouting system.

The ground grouting system of the Sitai Coal Mine is presented in Fig 7. Three ground grouting stations were established. The 1# grouting station was used as the centralized grouting station. The corresponding borehole was connected to the 311 return air flow roadway. The slurry flow was controlled using the valves. Moreover, the grouting target of the 1# grouting station was 81103, 81105, and 81107 gob areas, presented in Fig 7. Considering the high concentration of harmful gases in the 81103 and the 81105 gob areas, the 2# and 3# scattered grouting stations were also set up. The corresponding boreholes were connected to the 81103 and the 81105 gob areas, respectively, to guarantee that the purpose of grouting could be reached.

### Sealing the ground fissures

**Fig 8:**
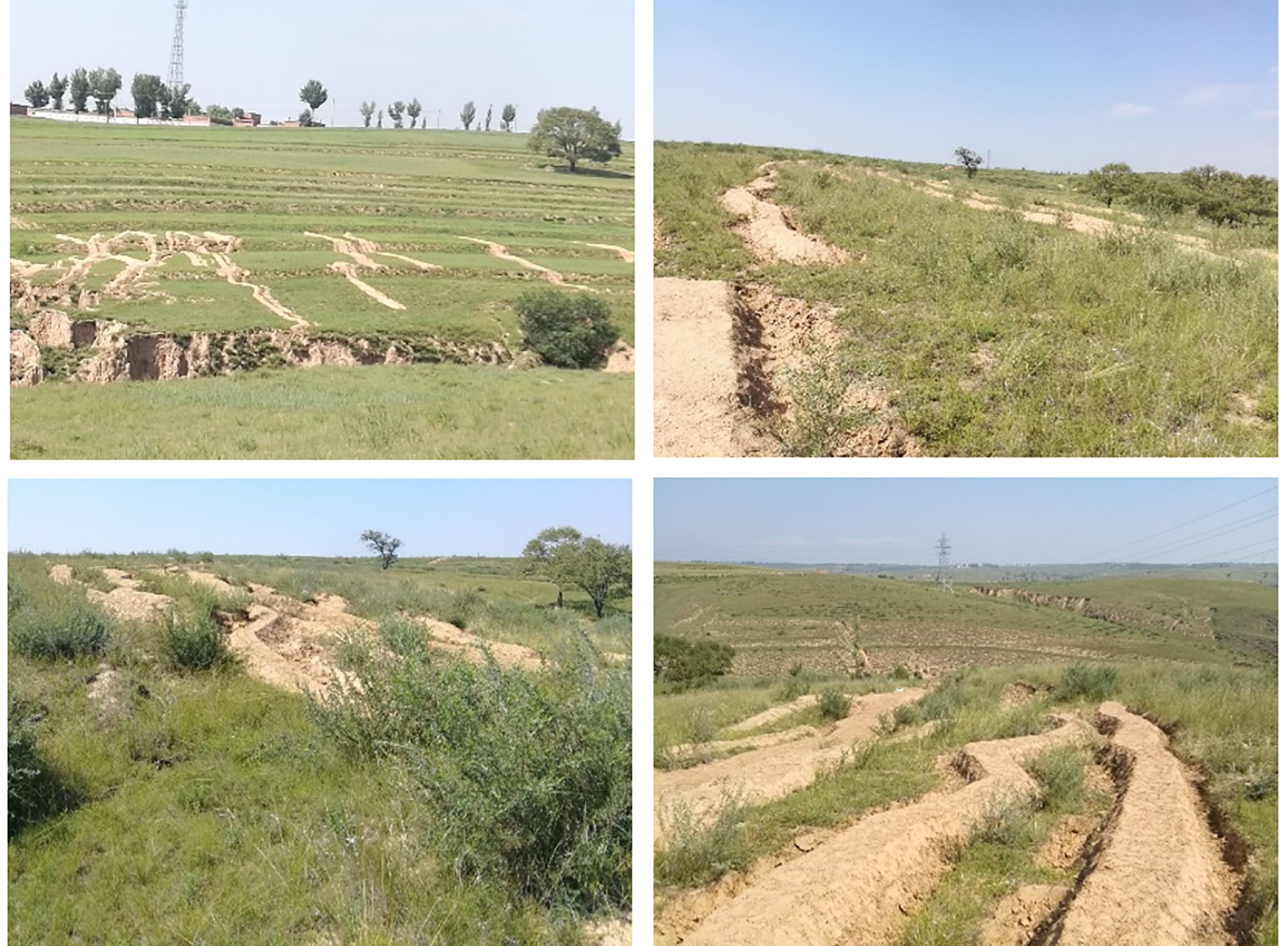
Ground fissures after loess filling.

In order to prevent the ground fissures from providing air leakage passages for the oxygen to enter the underground to promote coal oxidation, 12 fissures on the ground of the working face were sealed and loess was adopted as the sealing material. In the center of the ground fissure, the groove was first dug out, displaying a width of at least 2m and a depth of at least 3m. The loess around the fissure was consequently delivered to fill the groove, while water was sprinkled to the loess layer surface each time a loess layer of approximately 0.5m in thickness was completed. Finally, the loess was compacted using forklifts. The ground fissures after loess filling are presented in Fig 8.

## Field application results

The pressure balance measurement was carried out on the third day of mining, whereas the slurry grouting and fracture filling methods were conducted on the 20th day of mining.

**Fig 9:**
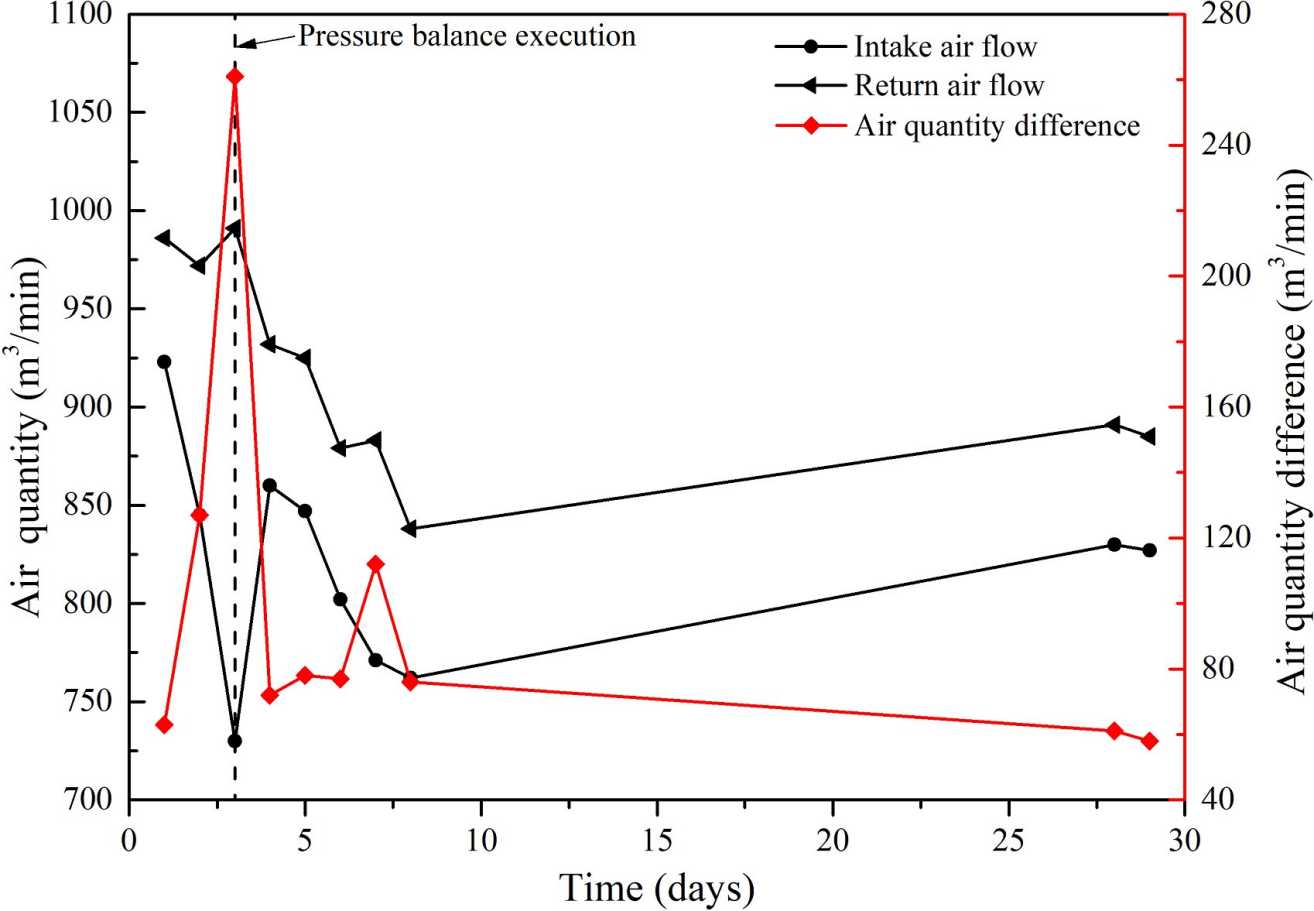
Air quantity variation of intake and return air flow in 81107 working face prior to and following pressure balance execution.

Fig 9 presents the air quantity variation of the intake and return air flows in the 81107 working face prior to and following the pressure balance execution. The air quantity monitors were in the intake and return airflow roadways, 30 m away from the 311 return airflow roadway. Notably, the air leakage quantity was equal to the air quantity difference between the intake and the return air flow. Prior to pressure balance execution, the air leakage quantity continuously increased as the mining progressed, while the maximum air leakage quantity was 261 m^3^min^-1^. Following pressure balance execution in the working face, the air leakage quantity decreased rapidly, and the air leakage quantity was limited to below 80 m^3^min^-1^ most of the time, as presented in Fig 9. The latter results indicated that the proposed pressure balance measure could effectively control fresh air flowing into the goaf, to inhibit oxygen supply for the low-temperature oxidation of coal.

**Fig 10:**
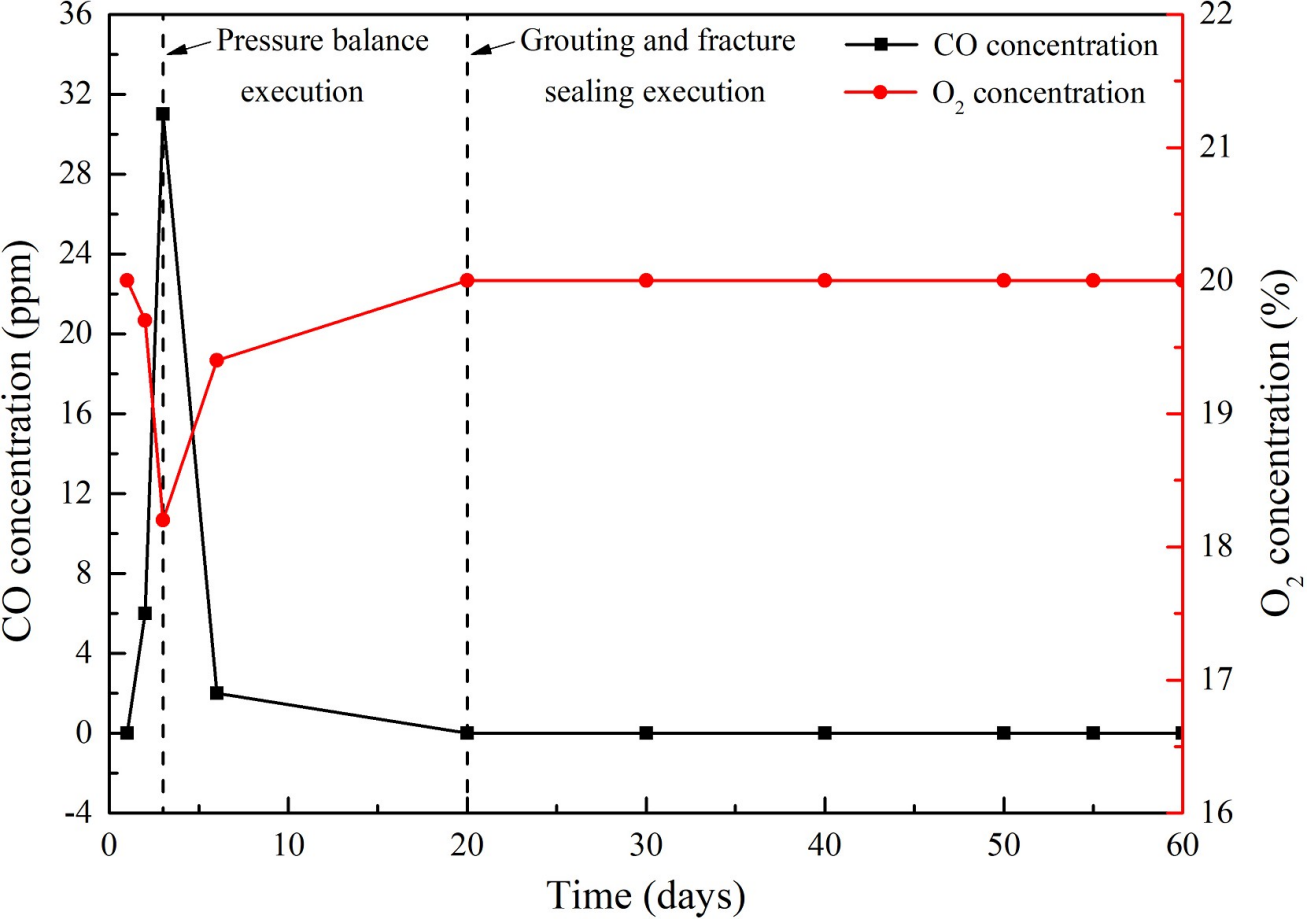
Variations of CO and O_2_ concentrations in the 81107 working face.

Fig 10 presents the variations of CO and O_2_ concentrations in the 81107 working face prior to and following the proposed comprehensive method. The CO and O_2_ monitors of working face were at the upper corner of 81107 working face. On the third day of mining, the concentration of CO reached 31ppm, exceeding the alarming concentration (24 ppm), while the O_2_ concentration was below 18.5%. Further, the pressure balance was adopted in the working face, which led to the decrease in the CO concentration to below 4 ppm. Moreover, the concentration of O_2_ continuously increased. The slurry grouting and fractures filling methods were conducted subsequently on the 20th day of mining. Yet the CO and O_2_ concentration variations in the working face were not apparent.

**Fig 11:**
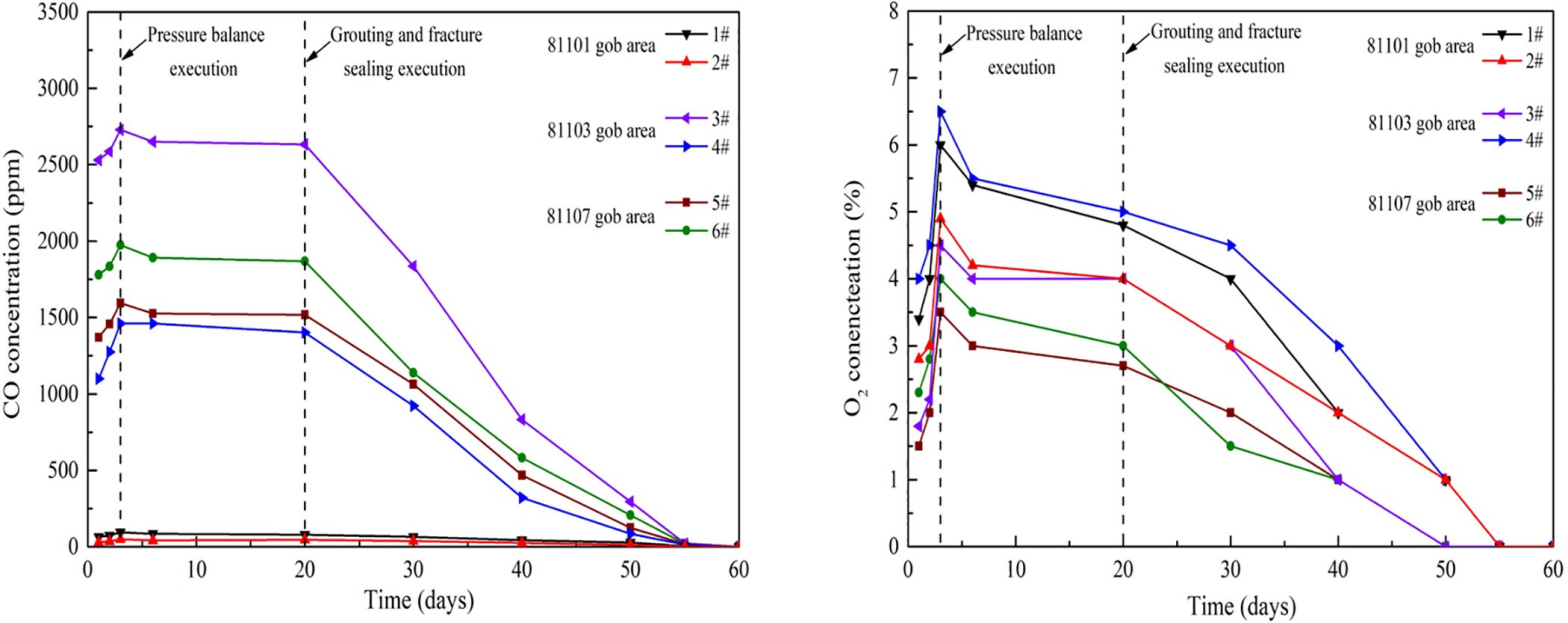
Variations of CO and O_2_ concentration in gob areas prior to and following proposed methods.

Fig 11 presents the variations of CO and O_2_ concentrations in gob areas. The gas samples were got in the gob, 2 m away from the upper corner of 81107 working face. Fig 11 demonstrates that prior to the application of prevention methods, the concentrations of CO and O_2_ in the goafs were high and continuously increased. Subsequent to pressure balance measurements, the CO and O_2_ concentrations of goafs decreased at a low speed initially and became stable within a certain range. Owing to the high areas of goafs, the pressure balance could not entirely control the harmful gases, while CO of high concentration existed in certain goafs, as presented in Fig 11. Following slurry grouting and fracture filling methods, the concentrations of CO and O_2_ in the goafs rapidly decreased, finally dropping to 0.

Prior to and following ground grouting method, the parameters of the gas in the boreholes were measured. The results are listed in Table 2. The results proved that following slurry grouting, the concentrations of CO and O_2_ in the goafs decreased to 0.

**Table 2:** Parameters of the gas in the 81105 gob borehole prior to and following ground grouting.

Gas	CH_4_ (%)	CO_2_ (%)	CO (ppm)	O_2_ (%)	Temperature (°C)	Pressure (Pa)
**Prior to ground grouting**	4.5	2.0	4800	13	13	1235.63
**Following ground grouting**	7.0	3.0	0	0	13	1274.86

The aforementioned results demonstrated that the pressure balance could effectively inhibit the gas exchange among the working face and the goafs, reducing the fresh air flow into the goafs, as well as decrease the O_2_ concentrations in the goafs and CO concentrations in the working face. The slurry grouting method could effectively overflow the goafs, as well as inhibit the coal oxidation and spontaneous combustion through the CO and O_2_ concentrations reduction in the goafs. The method of fracture filling could reduce the air leakage from the ground fissures and guarantee the goaf sealing. Subsequently to the proposed comprehensive methods adoption, at the working face, a portion of produced coal was recovered, and the risk of spontaneous combustion in the goafs was eliminated.

## Economic analysis

Table 3 lists the cost of comprehensive method.

**Table 3:** Cost of comprehensive method.

Component	Cost ($)
**pressure balance method**	72,580
**ground grouting**	64,484
**Cost of ground fissures sealing**	8,991.8
**Others**	500
**Sum**	146,555.8

The size of 81107 working face is 180*m*×607.5*m*, while the average thickness of the coal seam is 1.95m and the recovery rate is 0.95. The normal profit of coal is $17.4/t.

180×607.5×1.95×1.35×0.95=273,470.68 (1)

Thus 2.73×10^5^ ton coal could be extracted.

146,555.8÷273,470.68=0.54 (2)

The extra cost of comprehensive method is $0.54/t.

(17.4‑0.54)×273,470.68=4,610,715.66 (3)

The profit of 81107 working face is about $4.61×10^6^.

The working face shutdown because of the exacerbated risk of spontaneous combustion of coal; therefore, the proposed comprehensive method is necessary. The comprehensive method costs $1.47×10^5^, and leads to profit of $4.61×10^6^. Execution of the comprehensive method is thus economically reasonable.

## Conclusions

In this study, the analysis of mining-induced air leakage passages was presented, while the method to prevent spontaneous combustion of coal in longwall gob areas with complex air leakages was proposed. The field engineering practice was carried out in the Sitai Coal Mine in China.

The analysis results of air leakage passages indicated that two major air leakage passages existed affecting the production at the working face, which had the characteristics of high quantity and complex distribution: 1. Air leakage passages connecting the ground; and 2. Air leakage passages connecting the working face and the adjacent mine.

A comprehensive method, which combined pressure balance, grouting injection and filling ground fissures, was applied in the Sitai Coal Mine. First, the pressure balance was used in the working face, and then slurry grouting of the goafs was adopted. Furthermore, the ground fissures were filled with loess. Field application results indicated that the danger of spontaneous combustion of coal in the gob areas was eliminated. The successful application of the proposed method could provide references for the treatment of other coal mines.

## Supporting information

S1 File Supporting information file.xlsx. (XLSX)

## References

[1] Bai E, Guo W, Tan Y, Yang D. The analysis and application of granular backfill material to reduce surface subsidence in China's northwest coal mining area. PLoS ONE 2018;13(7): e0201112 10.1371/journal.pone.0201112 30036401 PMC6056034

[2] Hu G., Xu J., Ren T., Gu C., Qin W., Wang Z. Adjacent seam pressure-relief gas drainage technique based on ground movement for initial mining phase of longwall face. Int. J. Rock. Mech. Min. 2015;77: 237–45.

[3] Singh A.K., Singh M.P., Sharma M., Srivastava S.K. Microstructures and microtextures of natural cokes: A case study of heat-affected coking coals from the Jharia coalfield, India. Int. J. Coal. Geol. 2007;71: 153–175.

[4] Song Z., Kuenzer C. Coal fires in China over the last decade: a comprehensive review. Int. J. Coal. Geol. 2014;133: 72–99.

[5] Wang H., Chen C. Experimental study on greenhouse gas emissions caused by spontaneous coal combustion. Energ. Fuel. 2015;29: 5213–5221.

[6] Li X.C. China Coal Mine Safety Guidance. Beijing: Coal Industry Press; 1998.

[7] Qi G.S., Wang D.M., Zheng K.M., Xu J., Qi X.Y., Zhong X.X. Kinetics characteristics of coal low-temperature oxidation in oxygen-depleted air, J. Loss Prev. Process Ind. 2015;35: 224–231.

[8] Zhu H, Sheng K, Zhang Y, Fang S, Wu Y. The stage analysis and countermeasures of coal spontaneous combustion based on “five stages” division. PLoS ONE 2018;13(8): e0202724 10.1371/journal.pone.0202724 30138357 PMC6107200

[9] Wang S.F., Li X.B., Wang D.M. Mining-induced void distribution and application in the hydro-thermal investigation and control of an underground coal fire: A case study. Process Saf. Environ. Prot. 2016;102: 734–756.

[10] Qian M., Shi P., Xu J. Underground Pressure and Strata Control. Xuzhou: China University of Mining and Technology Press; 2010.

[11] Li X.B. Rock Dynamics Fundamentals and Applications. Beijing: Science Press; 2014.

[12] Yang J, Yu X, Yang Y, Yang Z. Physical simulation and theoretical evolution for ground fissures triggered by underground coal mining. PLoS ONE 2018;13(3): e0192886 10.1371/journal.pone.0192886 29513703 PMC5841665

[13] Xu X.L., Zhang N., Tian S.C. Mining-induced movement properties and fissure time-space evolution law in overlying strata. Int. J. Min. Sci. Technol. 2012;22: 817–820.

[14] Singh A.K., Singh R.V., Singh M.P., Chandra H., Shukla N.K. Mine fire gas indices and their application to Indian underground coal mine fires. Int. J. Coal. Geol. 2007;69: 192–204.

[15] Lu P., Liao G.X., Sun J.H., Li P.D. Experimental research on index gas of the coal spontaneous at low-temperature stage. J. Loss Prev. Process Ind. 2004;17: 243–247.

[16] Zhang J., Guan H., Cao D. Underground Coal Fires in China: Origin, Detection, Fire-Fighting, and Prevention Beijing: Coal Industry Press; 2008.

[17] Carras J.N., Young B.C. Self-heating of coal and related materials: models, application and test methods. Prog. Energy Combust. Sci. 1994;20: 1–15.

[18] Wolf K.H., Bruining H. Modelling the interaction between underground coal fires and their roof rocks. Fuel. 2007;86: 2761–2777.

[19] B Zhou F., Ren W.X., Wang D.M. Song T.L. Application of three-phase foam to fight an extraordinarily serious coal mine fire. Int. J. Coal Geol. 2006;67: 95–100.

[20] Colaizzi G.J. Prevention, control and/or extinguishment of coal seam fires using cellular grout. Int. J. Coal Geol. 2004;59: 75–81.

[21] Liang Y.T., Wang S.G. Prediction of coal mine goaf self-heating with fluid dynamics in porous media. Fire Saf. J. 2017;87: 49–56.

[22] Lu Y., Qin B.T. Identification and control of spontaneous combustion of coal pillars: a case study in the Qianyingzi Mine, China. Nat Hazards. 2015;75: 2683–2697.

[23] Brune J.F., Saki S.A. Prevention of gob ignitions and explosions in longwall mining using dynamic seals. Int. J. Min. Sci. Technol. 2017;27: 999–1003.

[24] Wang X., Hao Y.G., Song X.M., Ma X. Research on control of large area air leakage in gob area of Nantun coal mine. Safety in Coal Mines (China). 2011;42: 110–113.

[25] Palchik V. Formation of fractured zones in overburden due to longwall mining. Environ. Geol. 2003;44: 28–38.

[26] Wang S.F, Li X.B, Wang D.M. Void fraction distribution in overburden disturbed by longwall mining of coal. Environ. Earth Sci. 2016;75: 151.

[27] Sang S.X, Xu H.J, Fang L.C, Li G.J, Huang H.Z. Stress relief coalbed methane drainage by surface vertical wells in China. Int J Coal Geol. 2010;82: 196–203.

[28] Palchik V. Localization of mining-induced horizontal fractures along rock layer interfaces in overburden: field measurements and prediction. Environ Geol. 2005;48 (1): 68–80.

